# Neuroanatomy of post-stroke depression: the association between symptom clusters and lesion location

**DOI:** 10.1093/braincomms/fcad275

**Published:** 2023-10-25

**Authors:** Sebastian Krick, Janusz L Koob, Sylvia Latarnik, Lukas J Volz, Gereon R Fink, Christian Grefkes, Anne K Rehme

**Affiliations:** Department of Neurology, University Hospital Cologne, Cologne 50937, Germany; Department of Neurology, University Hospital Cologne, Cologne 50937, Germany; Department of Neurology, University Hospital Cologne, Cologne 50937, Germany; Department of Neurology, University Hospital Cologne, Cologne 50937, Germany; Department of Neurology, University Hospital Cologne, Cologne 50937, Germany; Institute of Neuroscience and Medicine, Cognitive Neuroscience (INM-3), Forschungszentrum Jülich, Jülich 52425, Germany; Department of Neurology, University Hospital Cologne, Cologne 50937, Germany; Institute of Neuroscience and Medicine, Cognitive Neuroscience (INM-3), Forschungszentrum Jülich, Jülich 52425, Germany; Department of Neurology, Goethe University Hospital Frankfurt, Frankfurt am Main 60528, Germany; Department of Neurology, University Hospital Cologne, Cologne 50937, Germany

**Keywords:** large-scale, MADRS, SVR-LSM, multivariate, neural substrates

## Abstract

Post-stroke depression affects about 30% of stroke patients and often hampers functional recovery. The diagnosis of depression encompasses heterogeneous symptoms at emotional, motivational, cognitive, behavioural or somatic levels. Evidence indicates that depression is caused by disruption of bio-aminergic fibre tracts between prefrontal and limbic or striatal brain regions comprising different functional networks. Voxel-based lesion–symptom mapping studies reported discrepant findings regarding the association between infarct locations and depression. Inconsistencies may be due to the usage of sum scores, thereby mixing different symptoms of depression. In this cross-sectional study, we used multivariate support vector regression for lesion–symptom mapping to identify regions significantly involved in distinct depressive symptom domains and global depression. MRI lesion data were included from 200 patients with acute first-ever ischaemic stroke (mean 0.9 ± 1.5 days of post-stroke). The Montgomery–Åsberg Depression Rating interview assessed depression severity in five symptom domains encompassing motivational, emotional and cognitive symptoms deficits, anxiety and somatic symptoms and was examined 8.4 days of post-stroke (±4.3). We found that global depression severity, irrespective of individual symptom domains, was primarily linked to right hemispheric lesions in the dorsolateral prefrontal cortex and inferior frontal gyrus. In contrast, when considering distinct symptom domains individually, the analyses yielded much more sensitive results in regions where the correlations with the global depression score yielded no effects. Accordingly, motivational deficits were associated with lesions in orbitofrontal cortex, dorsolateral prefrontal cortex, pre- and post-central gyri and basal ganglia, including putamen and pallidum. Lesions affecting the dorsal thalamus, anterior insula and somatosensory cortex were significantly associated with emotional symptoms such as sadness. Damage to the dorsolateral prefrontal cortex was associated with concentration deficits, cognitive symptoms of guilt and self-reproach. Furthermore, somatic symptoms, including loss of appetite and sleep disturbances, were linked to the insula, parietal operculum and amygdala lesions. Likewise, anxiety was associated with lesions impacting the central operculum, insula and inferior frontal gyrus. Interestingly, symptoms of anxiety were exclusively left hemispheric, whereas the lesion–symptom associations of the other domains were lateralized to the right hemisphere. In conclusion, this large-scale study shows that in acute stroke patients, differential post-stroke depression symptom domains are associated with specific structural correlates. Our findings extend existing concepts on the neural underpinnings of depressive symptoms, indicating that differential lesion patterns lead to distinct depressive symptoms in the first weeks of post-stroke. These findings may facilitate the development of personalized treatments to improve post-stroke rehabilitation.

## Teaser

Krick and Koob *et al*. demonstrate that different depression symptoms in acute stroke patients such as emotional, motivational, cognitive depressive, somatic and anxiety symptoms are linked to specific lesion locations. These findings reveal a more differential picture of lesion–symptom clusters compared with a depression sum score.

## Graphical Abstract

**Graphical Abstract:**
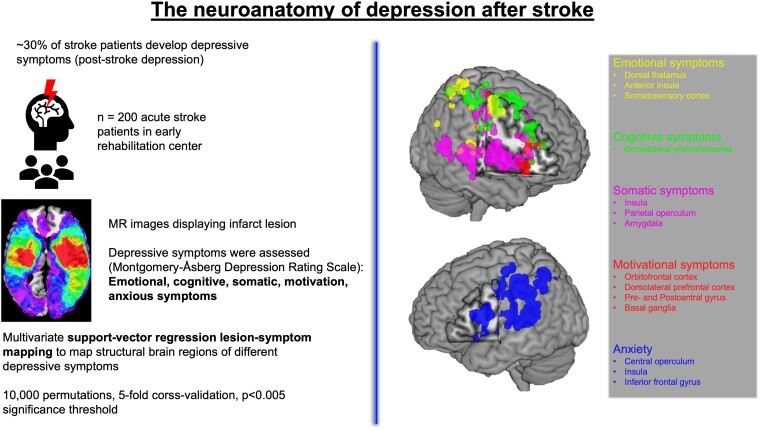

## Introduction

Stroke patients are at an increased risk of developing depressive symptoms, usually described as post-stroke depression (PSD) symptom complex.^1,2^ PSD is the most common neuropsychiatric consequence following stroke, with a prevalence of ∼30% of all patients.^3,4^ Notably, PSD symptoms hinder rehabilitation and functional outcome.^1,2^ Therefore, a better understanding of the neural mechanisms underlying PSD is critical for its prevention and the development of personalized treatment approaches.

According to the International Classification of Diseases, 10th revision, depression is based on heterogeneous symptomatology, affecting several domains of behaviour, including emotion, motivation, cognition, anxiety or somatic symptoms, e.g. sleep and appetite. Based on the monoamine hypothesis,^5,6^ the heterogeneity of depressive symptoms is linked to a dysfunction of ascending and descending bio-aminergic fibre tracts. In patients with major depression (MD), various studies using structural and functional MRI with different methodological approaches found significant alterations in frontal and prefrontal regions, including orbitofrontal cortex (OFC), dorsolateral prefrontal cortex (dlPFC), anterior cingulate cortex and subcortical structures, e.g. insula, putamen, caudate nucleus, thalamus, amygdala and hippocampus.^7-12^ These findings provide evidence for a ‘depression network’ within the human brain that contributes to depression severity and determines characteristic symptom domains.

In stroke patients, several studies aimed to determine whether specific lesion locations are associated with PSD. Many studies used univariate approaches such as voxel-based lesion–symptom mapping to investigate associations between infarct location and PSD.^13-15^ However, meta-analyses and reviews reported discrepant evidence, questioning a robust association between lesion sites or affected hemispheres and depressive symptoms.^2,16-20^ The reported inconsistencies across PSD studies may result from differences in samples, depression ratings and time since stroke onset. Moreover, methodological aspects of neuroimaging analyses may cause discrepant findings such as low spatial resolutions of lesion maps, false-positive results after multiple testing and neglecting voxel-wise dependencies in univariate voxel-based lesion–symptom mapping.^2,16,21^

Machine learning approaches address some of these limitations. Especially multivariate support-vector regression lesion–symptom mapping (SVR-LSM) allows us to compare all lesioned voxels simultaneously to predict continuous behaviour.^22^ SVR-LSM has been proven more sensitive and specific than classical mass univariate analyses in detecting lesion–symptom relationships.^23^ Particularly, the probability of lesions in neighbouring voxels is not random, as brain regions are organized in networks at both the structural and functional levels favouring multivariate approaches.^22,24-26^ Multivariate SVR-LSM has been used extensively in lesion–symptom studies investigating stroke patients with different functional impairments, including aphasia,^27^ cognitive impairment^28^ or visuo-spatial neglect,^29^ and identified specific regions in the frontal, temporal and parietal cortices to be associated with the respective clinical symptoms. In unipolar MD patients, multivariate machine learning analyses of structural and functional MRI data revealed altered anatomical corticolimbic networks associated with depressive symptoms.^30-32^

In contrast to the rich literature on the neural mechanisms underlying MD, few studies have thus far investigated putative structural correlates of PSD using multivariate lesion–symptom mapping methods, often with discrepant findings.^33-36^ Grajny *et al.*^33^ found lesions in dlPFC to be associated with higher levels of depression in chronic stroke patients, whereas Weaver *et al.*^34^ identified the right amygdala and right ventral pallidum as regions structurally linked to PSD in ischaemic stroke patients (<1-year post-stroke). Likewise, in chronic patients with focal brain lesions, Trapp *et al.*^35^ found a bilateral insula and dlPFC association with depression. Conversely, Sutoko *et al.*^36^ assessed acute ischaemic stroke patients and found lesions in the right Rolandic operculum linked to apathy, anxiety, perceived stress and depression post-stroke.

A critical reason for diverging results in PSD lesion–symptom mapping studies may lie in the heterogeneity of symptoms that constitute the diagnosis of depression.^6,37^ Thus, patients with PSD presenting similar global depression sum scores may considerably differ in clinical phenotype and underlying lesion–symptom associations. It was recently suggested that neural substrates of PSD might be uncovered at the individual symptom level instead of using a sum score.^38,39^ For example, patients with somatic depression may suffer from different lesion locations than depressive patients with predominantly motivational or cognitive symptoms. Consequently, analysing structure–function relationships using only global depression scores will inevitably mix different symptom categories and hence contribute to the inconsistency of lesion–symptom associations in depression. Notably, such analyses are only feasible with sample sizes that allow accounting for the heterogeneity of symptoms encountered in PSD.

Therefore, we investigated a large sample (*n* = 200) of acute stroke patients to link different functional domains of PSD symptomatology to lesion location using multivariate SVR-LSM.^22,24^ To identify lesion networks that contribute to different domains of depression, we built symptom domains of the Montgomery–Åsberg Depression Rating Scale (MADRS) interview,^40^ based on a conceptual–empirical approach according to the International Classification of Diseases, 10th revision criteria and internal psychological expertise. The resulting symptom domains consisted of motivational deficits, emotional symptoms, cognitive deficits, somatic symptoms and anxiety. To substantiate our results, we further computed five factors based on the MADRS items using principal component analysis and performed identical SVR-LSM analyses based on this data-driven approach.

Following previous multivariate SVR-LSM findings, we hypothesized that stroke lesions in the left dlPFC and ventral basal ganglia are associated with more severe depression.^33,34^ Furthermore, an essential aim of the present study was to identify lesion locations linked to different behavioural domains of PSD for the first time. On the basis of the literature on neural structures in MD patients, we expected lesions in prefrontal regions, limbic or striatal systems and insula to be specifically associated with distinct symptom domains, such as cognitive deficits, emotional dysregulation, motivational deficits, including apathy, and somatic symptoms, e.g. sleep disturbances and loss of appetite.^41-45^

## Materials and methods

### Study sample

Patients included in this study were retrospectively chosen from records of inpatients admitted to the early rehabilitation programme of the University Hospital of Cologne between 2015 and 2021. This programme encompasses medical care and specialized early therapeutic interventions within the first 4 weeks post-stroke. According to the German Diagnosis-Related Groups system, admission to this programme requires a certain degree of impairment based on the Early Rehabilitation Barthel Index,^46^ i.e. a score of 25 or less, indicating severe dependence on support for activities of daily living.

All patient data were extracted from the hospital patient database. Inclusion criteria were as follows: first-ever ischaemic stroke, MRI scan, National Institutes of Health Stroke Scale (NIHSS)^47^ score and sufficient cognitive and verbal abilities to undergo a MADRS interview. Patients with haemorrhagic stroke, spinal ischaemia, drug abuse, antidepressant medication or previous neurological or psychiatric disorders based on past diagnoses and current medical records were excluded from the study. A total of 1496 patients were admitted to the early rehabilitation programme between 2015 and 2021. Two hundred twenty-eight patients met our inclusion criteria. For 28 patients, only global MADRS sum scores were available from the medical records, whereas individual item symptom scores were available for 200 patients included in this study. In terms of modelling voxel-wise lesion location in SVR-LSM, Sperber *et al.*^48^ suggested a sample size larger than 140 subjects to be optimal. Patient data collection and study protocol were approved by the local ethics committee of the University Hospital of Cologne under the guidelines of the Declaration of Helsinki (revised in 2008).

### Lesion mapping and pre-processing

MRI scans were assessed on the patient’s admission to the hospital on average 0.9 days (±1.5) after the stroke. Diffusion-weighted images and fluid-attenuated inversion recovery images were used to map the individual lesions. Three different clinical MRI scanners with similar voxel sizes were used. Exact MRI protocols and scan parameters are reported in the Supplementary material. Lesions were manually segmented using the patient’s diffusion-weighted image scans by qualified neurologists, psychologists and neuroscientists using MRIcron.^49^ Lesion drawings underwent quality control by a second reviewer. Diffusion-weighted image, fluid-attenuated inversion recovery and lesion masks were spatially normalized to a standard Montreal Neurological Institute template (1 × 1 × 1 mm) using the unified segmentation approach^50^ with masked lesions in SPM12 (https://www.fil.ion.ucl.ac.uk/spm) implemented in MATLAB R2020a (The MathWorks Inc., Natick, MA, USA) and FMRIB Software Library. Note that unlike many other lesion–symptom mapping studies in stroke research, lesions were not systematically flipped to a particular hemisphere; i.e. information on inter-hemispheric differences in lesion location was preserved. Final pre-processing results were manually checked to ensure accurate co-registration and normalization of lesions.

### Lesion–symptom mapping

A MATLAB-based toolbox was used for multivariate lesion–symptom mapping,^24^ which is based on the SVR-LSM implementation introduced by Zhang *et al.*^22^ SVR is a special case of support vector machines, which are employed to solve binary classification problems, e.g. whether a disease is either present or absent.^51,52^ In contrast, SVR allows the prediction of continuous variables based on the lesion status of multiple voxels. The toolbox used for this study consists of an epsilon SVR with a non-linear Gaussian radial basis function kernel. All analyses were conducted using MATLAB R2020a on a high-throughput computing cluster of the Forschungszentrum Jülich (https://www.fz-juelich.de/inm/inm-7).

Controlling for lesion volume is essential in lesion–symptom mapping because patients with larger lesions tend to show more significant deficits.^24^ Thus, after correcting both the behavioural scores and the lesioned voxels for lesion volume, the interpretation of SVR-LSM results allows answering questions about whether the behaviour of interest is more strongly related to lesions in a particular brain area relative to all other brain regions rather than a mere correlative interpretation of whether lesions are associated with the behaviour of interest.^24^ Therefore, lesion volume was regressed from both the lesion maps and the behavioural variables for all SVR-LSM analyses. Stroke severity, as assessed by the NIHSS, age and sex were used as confound regressors. Of note, mild cognitive deficits as a symptom of both stroke and depression were difficult to disentangle and may still represent a potential confounder. A minimum lesion threshold of five lesions per voxel was used to ensure sufficient lesion overlap. The analysis design is one-tailed. Thus, the analyses were set to be negatively tailed based on the assumption that lesion presence was associated with higher MADRS scores indicating more severe depressive symptoms.

For model estimation, 5-fold cross-validation was used. Statistical significance was determined by a non-parametric approach using 10 000 permutations. A voxel was considered significant when passing a threshold of *P* < 0.005. Final permutation-based voxel-wise thresholded *P*-maps were smoothed using a 2 mm isotropic Gaussian smoothing kernel in SPM 12 to reduce cluster independence of neighbouring lesion voxels. Classification of significant anatomical structures was performed using the Harvard–Oxford cortical and subcortical structural atlases as implemented in FMRIB Software Library.

### Assessment of depressive symptoms

The MADRS interview is an observer-rated semi-structured depression scale consisting of ten items, each scored on a scale from 0 to 6, evaluated by several detailed interview questions.^40^ It measures the severity of depressive symptoms based on the patient’s condition over the past week, with higher scores indicating more severe depression. The following items are part of the MADRS: (i) apparent sadness, (ii) reported sadness, (iii) inner tension, (iv) reduced sleep, (v) reduced appetite, (vi) concentration difficulties, (vii) lassitude, (viii) inability to feel, (ix) pessimistic thoughts and (x) suicidal thoughts. Importantly, each item was rated using detailed questions from a clinical rater based on published clinical guidelines.^53^ All patients received a standardized neuropsychological assessment as part of the early rehabilitation programme. In our sample, the MADRS interview was assessed on average 8.4 days (±4.3) post-stroke.

## Categorization of depressive symptoms

### Conceptual–empirical approach

To test whether individual symptoms of depression were differentially linked to brain lesion locations, we used depressive symptom domains based on detailed clinical questions for each item of the MADRS interview. In a conceptual–empirical approach, five symptom domains were formed, covering distinct aspects of depression. These were based on the sum of single items, which were content related to a specific behavioural domain as described in the International Classification of Diseases, 10th revision and based on the expertise of six clinical psychologists, resulting in a high agreement of Fleiss’ kappa = 0.847.^54^ Additionally, extensive previous literature search was done to assess symptom cluster structures of the MADRS and other depression scales to form content-related specific symptom domains in our study.^55^ Generally, there are high cross-correlations between specific depressive symptoms (Supplementary Table 1); therefore, single MADRS items do not reflect separate entities but overlap between different domains.^53^ MADRS items were assigned to a symptom domain, which most likely represents the specific item. The depressive symptom domains were as follows: ‘Motivational symptoms’ included the items ‘lassitude’ and ‘inability to feel’ with questions such as difficulties in getting started or slowness in initiating and maintaining everyday activities, apathy, reduced interest in surroundings or activities that usually give pleasure, and reduced adequate emotional processing. ‘Emotional symptoms’ included the items ‘apparent sadness’ and ‘reported sadness’, assessed by interview questions on sadness, depressed mood, low spirit, helplessness, according to intensity, duration and extent, as well as apparent despondency, gloom and despair, reflected in speech, facial expression and posture. ‘Cognitive symptoms’ consisted of the following items: ‘concentration difficulties’, ‘pessimistic thoughts’ and ‘suicidal thoughts’, including questions about concentration deficits, thoughts of guilt, inferiority, remorse and ruin as well as suicidal thoughts and attempts. ‘Somatic symptoms’ included the items ‘reduced sleep’ and ‘reduced appetite’, based on questions about reduced duration or depth of sleep and loss of appetite. ‘Anxiety’ included the item ‘inner tension’, defined by questions on ill-defined discomfort, edginess, inner turmoil, mental tension with panic, dread or anguish. The scores of the conceptual–empirical categorization are summarized in Table 1.

**Table 1:** Overview of the demographic and clinical characteristics of the study sample (*n* = 200)

	Value
**Demographics**	
Sex (female:male)	114:86
Age (years), mean (±SD)	72.99 (12.77)
Lesion side (right:left:bilateral)	104:81:15
Examination NIHSS post-stroke (days), mean (±SD)	3.40 (2.01)
Examination NIHSS post-stroke (days), range	0–14
Examination MADRS post-stroke (days), mean (±SD)	8.43 (4.26)
Examination MADRS post-stroke (days), range	1–22
Examination MADRS post MR (days), mean (±SD)	7.55 (4.24)
Thrombectomy (*n*)	53
Thrombolysis (*n*)	67
Lesioned voxels (cm^3^), mean (±SD)	33.58 (50.91)
Lesioned voxels (cm^3^), range	0.01–268.11
**Depressive symptom domains (MADRS)**	
Global sum score, mean (±SD)	9.11 (7.09)
Anxiety domain, mean (±SD)	1.10 (1.39)^a^
Somatic symptoms domain, mean (±SD)	1.44 (1.37)^a^
Emotional symptoms domain, mean (±SD)	1.24 (1.18)^a^
Cognitive symptoms domain, mean (±SD)	0.51 (0.71)^a^
Motivational symptoms domain, mean (±SD)	0.55 (0.89)^a^
**Global impairment**	
NIHSS, mean (±SD)	12.85 (4.56)

NIHSS, National Institute Health Stroke Scale; MADRS, Montgomery–Åsberg Depression Rating Scale. ^a^Note that for the depressive symptom domains, each domain was standardized based on the number of MADRS items included in this domain to allow for a direct clinical comparison between domains.

### Data-driven corroboration

In a data-driven approach, we aimed to further substantiate the conceptual–empirical categorization of five depression domains by computing a factor analysis in SPSS 28 (IBM Corp, Armonk, NY, USA) using the 10 MADRS item scores of the patient sample. Therefore, a principal component analysis was performed with an oblique rotation procedure to obtain a realistic representation of the correlative structure underlying depression factors.^56,57^ We entered a fixed number of five factors for factor extraction derived from the five conceptual–empirical symptom domains, applying the total variance explained extraction criterion. This criterion suggests extracting factors until a specific threshold of explained cumulative variance is reached, which is usually set between 70% and 90%.^58,59^ Extracting five factors resulted in a cumulative explained variance of 72%, which corresponds to an Eigenvalue threshold of 0.8 in our data (Supplementary Tables 2 and 3 and Supplementary Fig. 3). Factor score coefficients were estimated and used as behavioural input variables in SVR-LSM. All SVR-LSM analyses were carried out identically to the analyses of the conceptual–empirical domains. The clinical designation of the factors, factor loadings, eigenvalues, explained variance and the corresponding SVR-LSM analyses and significant voxels of cluster regions are reported in the Supplementary material.

### Statistical analysis

Spearman correlations for ordinal-scaled variables were used to assess sample associations between the MADRS scores (sum score and symptom domains of the conceptual–empirical approach) and NIHSS, age and lesion volume in SPSS 28. To assess differences in depressive symptoms between sexes, we performed a one-way ANOVA. False discovery rate correction for multiple testing was applied for all analyses.^60^ Importantly, to investigate the potential influence of functional impairment on depression most accurately, we applied the individual NIHSS, which was assessed closest to the MADRS interview for the statistical analyses and SVR-LSM. The MADRS interview [8.4 days (±4.3) of post-stroke] was assessed always after potential interventions like thrombectomy, thrombolysis or tissue plasminogen activator medication. The NIHSS scores were assessed 3.40 days (±2.01, range: 0–14) post-stroke. Thus, NIHSS scores are only indirectly related to the initial level of impairment assessed upon admission and whether a patient received immediate treatment.

A total of 11 multivariate SVR-LSM analyses were carried out, including the global MADRS score, the conceptual–empirical scores and the data-driven factor coefficients for five symptom domains as behavioural variables.

The Dice coefficient (DC) was calculated to quantify the similarity of spatial lesion overlap of *P*-maps of the conceptual–empirical approach and the corresponding data-driven approach.^61^ We used the ‘fslstats’ and ‘fslmaths’ commands implemented in FMRIB Software Library to compute overlapping voxels between each of the five symptom domains and clinically corresponding factors (see Supplementary material) by multiplying both maps with each other. To calculate the DC, we used the formula DC (X,Y) = (2|X ∩ Y|)/(|X| + |Y|), where |X| is the total number of significant voxels in lesion map X and |Y| the total number of significant voxels in lesion map Y. |X ∩ Y| indicates the number of overlapping voxels of both lesion maps. DC was computed for each of the five pairs of symptom domains and corresponding factors. The coefficient ranges from 0 to 1, with 0 indicating no overlap and 1 indicating perfect overlap (low: 0–0.19; low-moderate: 0.20–0.39; moderate: 0.40–0.59; moderate-high: 0.60–0.79; and high: 0.80–1.00).^62^

## Results

Clinical and demographic data are shown in Table 1. MADRS scores were distributed in the sample as follows: 89 patients (44.5%) showed no depressive symptoms, 95 patients (47.5%) were mildly depressed, 15 patients (7.5%) were moderately depressed, and one patient (0.5%) showed severe depressive symptoms.^63^ Note that due to the admission criteria for entering early rehabilitation treatment, patients had a more significant neurological impairment than the general stroke population,^64^ which is also evident from the relatively high mean NIHSS score of 12.85 (±4.56) (Table 1).

Correlational analyses between the depression sum score, depressive domains and the one-way ANOVA comparing depression scores between sexes revealed no associations with age, lesion volume, sex or stroke severity (NIHSS), respectively (all *P* > 0.384; false discovery rate corrected). This suggests that symptoms of depression were not solely explained by the amount of stroke-induced functional impairments.

### SVR-LSM results

**Figure 1:**
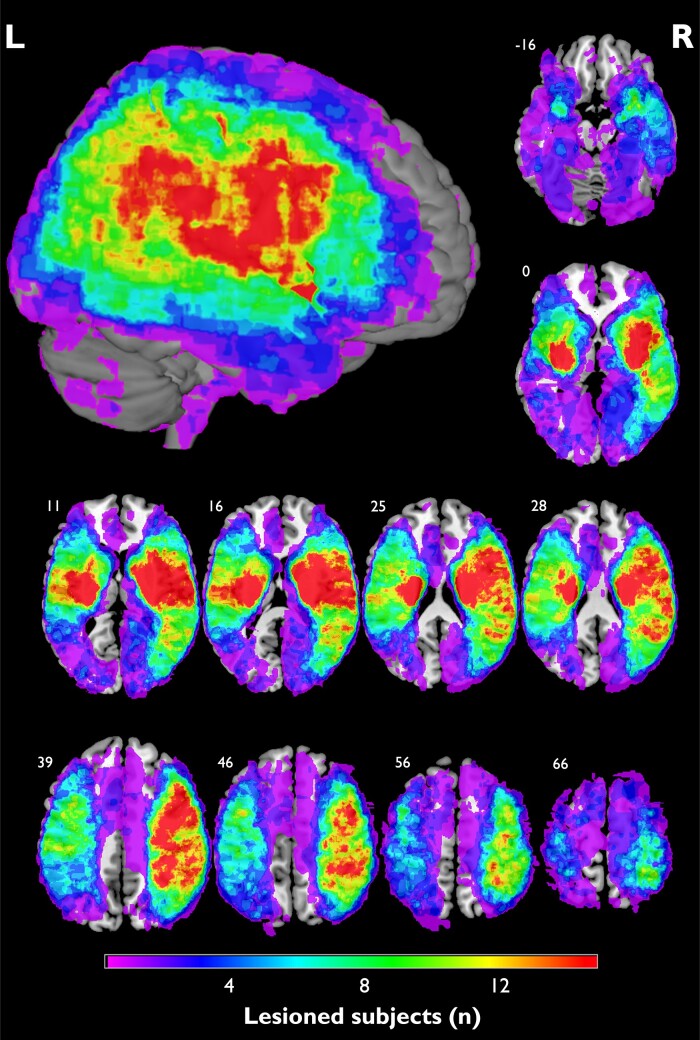
**Lesion coverage map.** Overlap map of normalized lesions from patients included in the analysis (*n* = 200). Coordinates indicate the corresponding *z*-value in the Montreal Neurological Institute space. Colours indicate the amount of lesion overlap. The highest overlap was seen at the right putamen (*n* = 41). Please note that small overlap into ventricles is due to co-registration for display purposes in MRIcron. L, left; R, right.

The average lesion volume was 33.58 cm^3^ (±50.91 cm^3^; range: 0.01–268.11 cm^3^). Figure 1 shows the lesion coverage for the entire patient sample (*n* = 200). The region with the highest overlap was at the right putamen (*n* = 41, 20.5%). High lesion coverage of the left and right hemispheres was observed, except regions surrounding frontal and occipital poles, cingulate gyrus and precuneus. Eighty-one patients had lesions in the left hemisphere, 104 patients had right hemispheric lesions, and 15 had bilateral damage. A lesion overlap map of *n* ≥ 5 patients included in the SVR-LSM is displayed in Supplementary Fig. 1. Please note that the MADRS interview was not assessed in patients who were unable to comprehend and adequately respond to an interview. Thus, as aphasia mostly results from left-sided lesions, patients with severe aphasia were not included in our sample. Therefore, the imbalance of right and left hemispheric lesions may be caused by the inability to assess severely aphasic stroke patients in a formal interview. Similarly, patients with severe cognitive impairment were excluded from MADRS interviews. Of note, cognitive dysfunction might display a potential confounder in the analysis of depression symptoms in the acute stage post-stroke. Although patients with severe cognitive dysfunctions were not included in our sample, it is still possible that mild cognitive decline has an impact on our findings.

**Figure 2:**
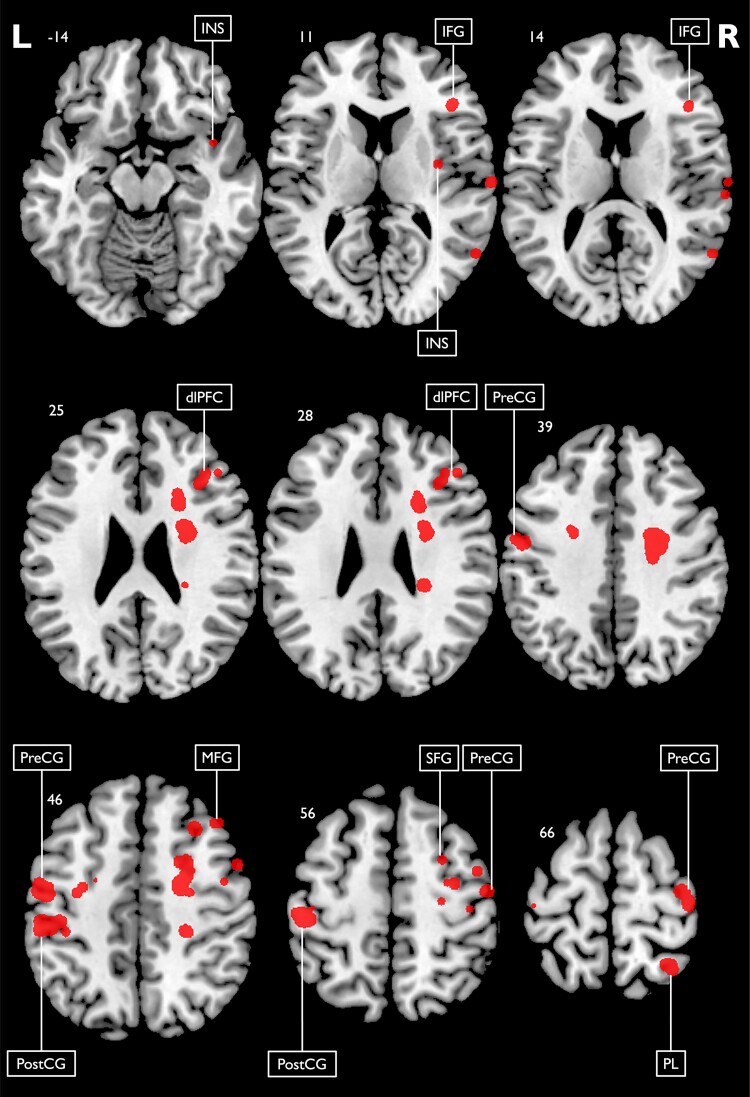
**SVR-LSM results of the global depression sum score.** SVR-LSM results and lesion locations associated with the global MADRS score with a voxel-wise significance threshold set to *P* < 0.005 (*n* = 200). Results were smoothed using a 2 mm isotropic Gaussian smoothing filter. Classification of anatomical structures was performed using the Harvard–Oxford cortical and subcortical structural atlases. Coordinates indicate the corresponding *z*-value in the Montreal Neurological Institute space. Predominant clusters are labelled. dlPFC, dorsolateral prefrontal cortex; IFG, inferior frontal gyrus; INS, insula; L, left; MFG, middle frontal gyrus; PL, parietal lobe; PostCG, post-central gyrus; PreCG, pre-central gyrus; R, right; SFG, superior frontal gyrus.

**Figure 3:**
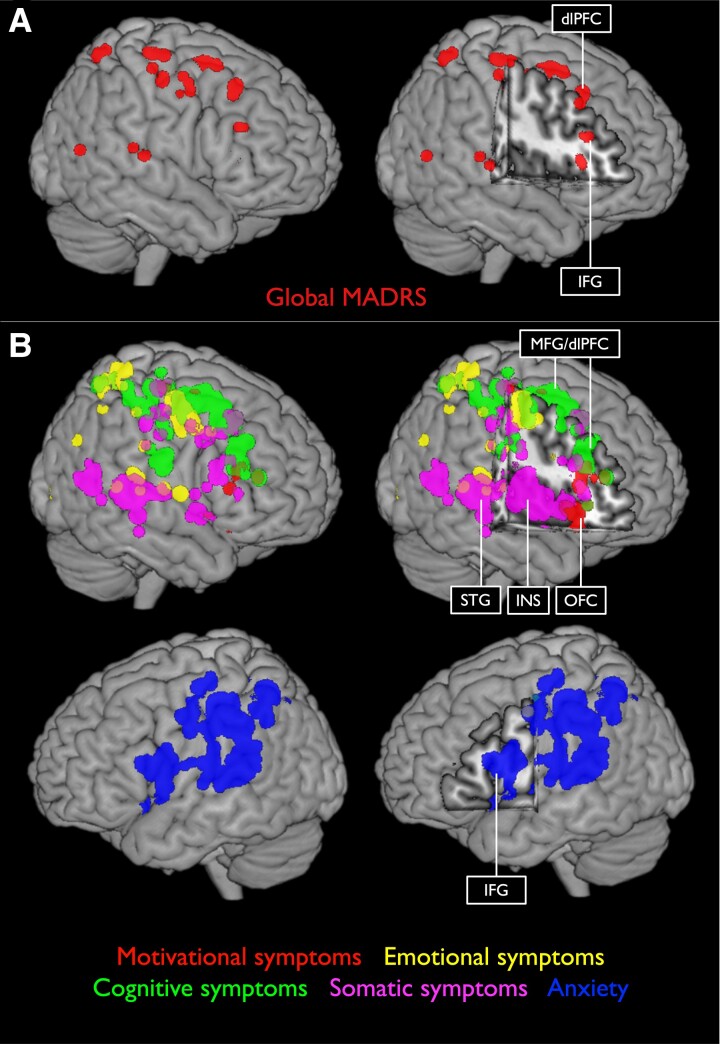
**3D renderings of SVR-LSM results.** 3D renderings of SVR-LSM results displaying the global MADRS score (**A**) and the five symptom domains based on the conceptual–empirical classification with a voxel-wise threshold set to *P* < 0.005 (*n* = 200) (**B**). dlPFC, dorsolateral prefrontal cortex; IFG, inferior frontal gyrus; INS, insula; MFG, middle frontal gyrus; OFC, orbitofrontal cortex; STG, superior temporal gyrus.

SVR-LSM results revealed that the MADRS sum score was specifically related to lesions in dlPFC and inferior frontal gyrus (IFG). Thus, patients with lesions in these locations indicated higher depression scores. Results are displayed in Figs 2 and 3A.

**Figure 4:**
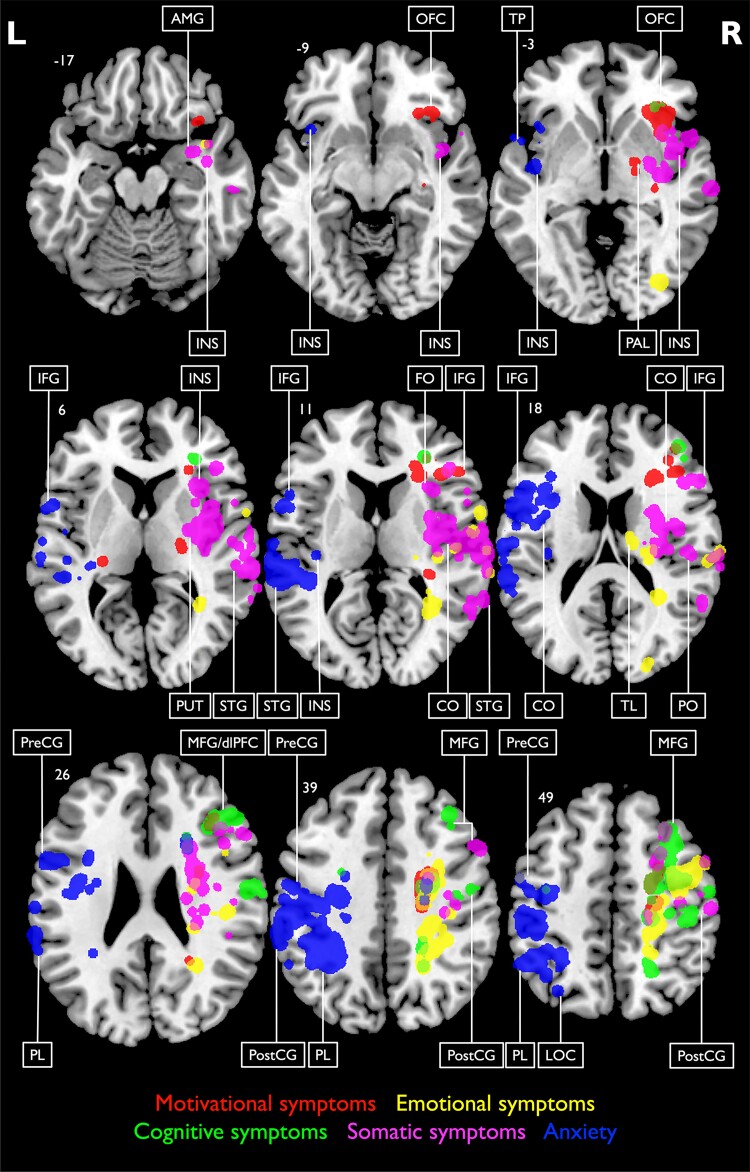
**SVR-LSM results and lesion location associations of depressive symptom domains.** SVR-LSM results and lesion location associations of depressive symptom domains based on the conceptual–empirical classification with a voxel-wise threshold set to *P* < 0.005 (*n* = 200). Results were smoothed using a 2 mm isotropic Gaussian smoothing filter. Coordinates indicate the corresponding *z*-value in the Montreal Neurological Institute space. Specific symptom domains are labelled by different colours. Classification of anatomical structures was performed using the Harvard–Oxford cortical and subcortical structural atlases. Predominant clusters are labelled. AMG, amygdala; CO, central operculum; dlPFC, dorsolateral prefrontal cortex; FO, frontal operculum; IFG, inferior frontal gyrus; INS, insula; L, left; LOC, lateral occipital cortex; MFG, middle frontal gyrus; OFC, orbitofrontal cortex; PAL, pallidum; PL, parietal lobe; PO, parietal operculum; PostCG, post-central gyrus; PreCG, pre-central gyrus; PUT, putamen; R, right; STG, superior temporal gyrus; TL, thalamus; TP, temporal pole.

We analysed lesion–symptom relationships for the five different domains of depression defined by the conceptual–empirical criteria (Figs 3B and 4; Table 2). Motivational deficits showed lesion associations with the OFC, dlPFC, pre- and post-central gyri and basal ganglia, including putamen and pallidum. Emotional symptoms were significantly related to lesions in the dorsal thalamus, anterior insula and somatosensory cortex. Cognitive symptoms were primarily associated with damage to dlPFC. Additionally, somatic symptoms were linked to insula, parietal operculum and amygdala lesions, whereas symptoms of anxiety were associated with lesions in the central operculum, insula and IFG (see Supplementary Fig. 2 for individual maps). In summary, SVR-LSM results of the depressive symptom domains revealed a differential and precise picture with lesion–symptom associations not detected in the SVR-LSM analysis of the MADRS sum score.

**Table 2:** SVR-LSM results of the global MADRS score and the conceptual–empirical classification of depressive symptom domains

Brain region	Global MADRS	Motivational symptoms	Emotional symptoms	Cognitive symptoms	Somatic symptoms	Anxiety
Inferior frontal gyrus	R	R			R	L
Middle frontal gyrus	R (dlPFC)	R (dlPFC)	R	R (dlPFC)	R (dlPFC)	
Superior frontal gyrus	R	R		R	R	
Insula	R	R (a)	R (av)		R	L
Pre-central gyrus	L/R	R	R	R	R	L
Post-central gyrus	L/R	R	R	R	R	L
Middle temporal gyrus					R	
Superior temporal gyrus	R		R		R	L
Inferior parietal lobe			R		R	L
Superior parietal lobe	R		R	R		L
Amygdala					R	
Frontal operculum					R	
Central operculum			R		R	L
Parietal operculum			R		R	L
Putamen		R			R	
Pallidum		R			R	
Temporal pole				R	R	
Thalamus			R (d)			
Orbitofrontal cortex		R		R		
Lateral occipital cortex						L
Pons						L

L(eft) and R(ight) indicate the hemisphere with significant clusters of voxels (*P* < 0.005) in a given brain region. Classification of anatomical structures was performed using the Harvard–Oxford cortical and subcortical structural atlases. a, anterior; av, anterior-ventral; d, dorsal; dlPFC, dorsolateral prefrontal cortex.

SVR-LSM results of the data-driven symptom classification yielded highly similar results and are reported in Supplementary Fig. 4 and Supplementary Table 4. As a measure of similarity, the DC yielded moderate-high overlap (0.64 ± 0.08), averaged across all analyses.^62^ For the respective symptom domains, the coefficients ranged between moderate and moderate-high similarities: motivational symptoms/Factor 5 DC = 0.52; emotional symptoms/Factor 3 DC = 0.64; cognitive symptoms/Factor 4 DC = 0.73; somatic symptoms/Factor 2 DC = 0.63; and anxiety/Factor 1 DC = 0.69. See the Supplementary material for further contextual information on the comparison between the conceptual–empirical and data-driven SVR-LSM results.

## Discussion

We used large-scale multivariate lesion–symptom mapping to identify lesion patterns associated with distinct behavioural domains of depression in the acute stage post-stroke. We obtained a much more differential picture of the structural correlates underlying distinct depressive symptoms, than the usage of a sum score for depression. Importantly, by controlling for various covariates (neurological and psychiatric history, lesion volume, stroke severity, age and sex), SVR-LSM findings from our study are specifically related to the acute depressive symptomatology post-stroke, thereby providing further evidence that depressive symptoms may derive from lesions to specific brain areas than representing a mere adjustment disorder. Likewise, lesion–symptom associations were primarily independent of the categorization approach of standard MADRS depression interview scores using either conceptual–empirical or data-driven classification.

### Depression as a multi-dimensional syndrome

The finding that specific brain structures contribute to distinct domains of depression, including motivational, emotional and cognitive deficits as well as somatic symptoms and anxiety, enables a new taxonomy to further our understanding of depression in general. Additionally, by using a multivariate SVR-LSM approach with continuous behavioural scores, the sensitivity and robustness of lesion–symptom associations are increased compared with classical mass univariate LSM analyses.^22^ Furthermore, we observed symptom-specific hemispheric lateralization of brain–behaviour associations: Despite fewer left hemispheric lesions (Fig. 1; Table 1), we found that symptoms of anxiety were predominantly associated with left-lateralized lesions in both classification approaches. All other four symptom domains (emotional, somatic, motivational and cognitive) were associated with right hemispheric lesions. The role of lesion lateralization in PSD remains a topic of scientific debate. Several meta-analyses and reviews reported no significant influence,^16,18,20^ whereas some found left hemispheric lateralization,^17^ and others found right hemispheric lateralization but only in the sub-acute stage post-stroke.^19^ The present findings provide the first evidence that lesion lateralization in PSD might be symptom-specific. This discovery suggests that lateralization may only be revealed by considering PSD as a multi-dimensional disorder. This heterogeneous classification of depressive symptoms and associated neural substrates furthers our understanding of the mechanisms underlying the brain–behaviour relationship in PSD.

The results of the global depression score revealed no other lesion–symptom associations beyond the lesions in brain areas that were specifically related to different symptom domains of depression. The findings for the global depression score partially corroborate evidence based on previous multivariate lesion–symptom mapping studies in sub-acute and chronic stroke.^33-35^ In line with our results, structural lesions in dlPFC, amygdala and ventral pallidum were linked to more severe depression. In the following, neural correlates of individual depressive symptom domains will be discussed.

### Motivational symptoms

Motivational deficits were based on item questions such as difficulties in getting started or slowness in initiating and maintaining everyday activities, apathy, reduced interest and reduced adequate emotional processing.^40,53,63^ We found that pronounced motivational deficits were primarily related to damage in OFC, dlPFC, pre- and post-central gyri and basal ganglia, including putamen and pallidum. These regions constitute the human corticostriatal reward network, which sub-serves incentive motivational behaviour by transforming motivations and cognitions into actions.^6,65-67^ This motivational system may be differentiated into ventral, and dorsal corticostriatal networks, organized by reciprocal loops in a topographic manner to translate motivations into actions, regulate emotions and mediate goal-directed behaviour.^65,68^ Functional MRI activity in dlPFC and striatum has been reported to correlate with reduced incentive motivation in MD patients.^69,70^ Besides, reduced incentive motivation in stroke patients is affected by apathy post-stroke, resulting from damage to bilateral basal ganglia, including the ventral striatum.^71,72^ Our analyses revealed lesion–symptom associations in OFC and basal ganglia, which play a crucial role in the human corticostriatal reward system.^6,67^

### Emotional symptoms

More significant emotional symptoms of perceived and observed sadness, depressed mood, low spirit, helplessness, gloom and despair were linked to lesions in the anterior-ventral part of the insula, dorsal part of the thalamus and post-central gyrus. In a large-scale meta-analysis, the anterior-ventral insula was found to be relevant for emotion and empathy.^73^ The broad literature supports the finding that the insula, specifically the anterior part, is an essential correlate for socio-emotional stimulus processing.^74-79^ Furthermore, a structural MRI study by Tippett *et al.*^80^ observed that acute stroke patients with lesions in the right amygdala and right anterior insula performed significantly worse in facial emotion recognition tasks than patients with other lesion locations.

Further correlations were observed between emotional symptoms and lesions of the dorsal thalamus. The thalamus is seen as the gatekeeper to the cerebral cortex due to interconnections to various brain areas, including the insula, amygdala or frontal cortex, which contribute to attention, memory, consciousness, sleep, arousal and emotion.^81,82^ The thalamus may therefore not be primarily involved in the emotional symptoms of PSD, but lesions may influence its modulating role on connected areas.

Furthermore, emotional symptoms correlated with lesions in the post-central gyrus and parietal operculum, i.e. the primary somatosensory cortex (S-I) and secondary somatosensory cortex (S-II). One study found structural and functional S-I and S-II changes in patients with mental disorders including depression, anxiety and panic disorder.^83^ There is further evidence that the somatosensory cortex is involved in regulating emotions evoked by somatosensory stimuli by using strategies of attention direction in the context of social adequacy.^83^ Likewise, somatosensory representation in recognizing emotional states in facial expressions has previously been associated with damage to the right S-I, S-II and insula, even with the absence of lesions in primary visual brain areas.^84^

### Cognitive symptoms

The cognitive symptom domain included concentration deficits and different items referring to ‘mindsets’ consisting of thoughts of guilt, inferiority, remorse and ruin, as well as suicidal thoughts. It was primarily correlated with more pronounced lesions in large parts of the middle frontal gyrus, including dlPFC. The dlPFC is mainly involved in executive functions, including attentional processing and working memory for goal-directed actions.^85^ Therefore, dysfunction of dlPFC may severely affect cognitive and executive functions such as attentional processing and divided attention,^86^ which also results in concentration deficits in MD patients.^87,88^

Previous literature already showed that a reduction of grey matter tissue in dlPFC contributes to depressive symptoms in late-life depression.^89^ In line with the monoamine hypothesis of corticolimbic dysregulation, reduced functional connectivity of dlPFC, amygdala and anterior cingulate cortex is associated with impaired regulation of negative emotion processing based on enhanced processing of negative stimuli.^6,37,90-92^ Lesion–symptom mapping studies on PSD reported strong correlations between dlPFC lesions and global depression.^33^ Moreover, repetitive transcranial magnetic stimulation to dlPFC is an established approach for treating depression.^93-95^ Of note, a recent study including patients with several lesion aetiologies in five different data sets showed that instead of the lesion location itself, functional connectivity of lesions with left dlPFC was significantly related to depression.^14^ The authors concluded that dlPFC represents a connection hub for depressive symptoms and a target for interventions.

Our results at the acute stage post-stroke, together with previous literature, support the hypothesis that the dlPFC holds a critical role in depression^14,17,20,33,96,97^ and further extend these findings by specifying cognitive symptoms of depression, specifically concentration deficits, to be a dominant symptom in stroke patients with dlPFC damage.

### Somatic symptoms

We found somatic depressive symptoms like sleep disorders and loss of appetite were primarily associated with damage to the insula, parietal operculum, amygdala and parietal lobe. The posterior insula has been shown to play a role in integrating primary interoceptive signals with stronger emotionally salient information gradually represented by anterior insula, which was significantly associated with somatic symptoms in our SVR-LSM analysis.^98^ The amygdala is involved in encoding emotional valence from emotionally salient stimuli.^99^ Furthermore, the parietal operculum, i.e. S-II, and insula mediate gustatory and olfactory processing.^73,100^ There is broad evidence that not only interoceptive sensations like fatigue, hunger, pain or sexual drive but also heartbeat are disturbed in MD patients.^101-105^ These effects are mediated by reduced insula activation, probably via interconnections with primary and secondary somatosensory areas in the parietal cortex.^106,107^

Interestingly, a recent review found low-frequency repetitive transcranial magnetic stimulation over the right dlPFC or posterior parietal cortex to reduce sleep problems in patients with primary insomnia.^108^ Thus, depression may lead to misinterpretation of bodily signals to achieve homeostasis of somatic needs.^98,102,103^ The misinterpretation of bodily sensations may affect other behavioural dysfunctions in depression, including motivational deficits, emotional dysregulation or even alexithymia.^101,105,109^

### Anxiety

More significant anxiety, in particular inner tension such as discomfort, edginess, inner turmoil, panic, dread, anguish and loss of interest, was linked to lesions in the insula, IFG, central operculum, and parietal, temporal and occipital cortices. A recent study on ischaemic stroke patients suggested that post-stroke apathy, anxiety and depression were associated with damage to the central operculum.^36^ Previous studies in healthy subjects identified the insula as an essential neural correlate in mediating anxious traits.^101,110,111^ Specifically, the insula plays a crucial role in detecting differences between an expected and observed body state followed by increased anxious feelings, which leads to increased anxiety in anticipation of a future aversive body state.^101^ Likewise, a study examining stroke patients with frontal brain lesions found that structural abnormalities in the insula are closely related to elevated sensitivity to anxiety.^112^ Thus, the existing literature suggests a predominant role of the insular cortex in states of stress and anxiety associated with uncertain situations, overestimated potential adverse outcomes and risk-taking decision-making behaviour.^113-116^

SVR-LSM results further revealed significant clusters in IFG. Cha *et al.*^117^ found altered IFG dynamics linked to abnormal structural and functional prefrontal-limbic connectivity in clinically anxious individuals. The authors suggested that IFG plays a crucial role in modulating fear and anxiety in response to threats. Overall, lesions in the insula, IFG, central operculum and parietal cortex play a role in developing anxious symptoms in acute stroke patients. A recent review reported evidence for an ‘advanced fear network model’ including these brain areas and hypothesized that fronto-limbic dysregulation is induced via sensory modalities from temporal, parietal and occipital cortices.^118^ Sensory information is filtered by the thalamus, processed by the insula and further integrated into the fronto-limbic loop for cognitive and autonomic responses, including symptoms of anxiety.

### Limitations

Despite the strengths of our study, it is crucial to address some limitations. One pertains to the inclusion of acute stroke patients, which introduces a potential confounding factor due to the progressive evolution of lesions over time, especially in the first 24 h. Ischaemic penumbra or diaschisis, representing regions of brain tissue surrounding and functionally connected to the lesion site, may exert an influence on the symptoms experienced by patients.^119^ In our sample, the assessment of depressive symptoms occurred on average 8.43 days following the MR scan. It is important to note that during this interval, the ongoing evolution of the lesion might have impacted the manifestation of symptoms. In addition, it is important to note that the patient sample exhibited on average mild depressive symptoms (mean MADRS score of 9.1). This places patients only on the brink of meeting criteria for mild depression. The lack of individuals with more severe depressive symptoms warrants consideration of this potential influence. Furthermore, despite having a large patient sample, certain regions (such as those surrounding frontal and occipital poles, thalamus, cingulate gyrus or medial prefrontal gyrus) were excluded in the analysis due to insufficient lesion overlap and cannot be concluded about. Consequently, conclusions are restricted to included regions.

### Pathophysiology of PSD

PSD has been discussed as arising from a complex interplay of multi-dimensional biological, functional and psychosocial aspects.^21,120^ PSD severity and potential risk factors may vary considerably depending on the time post-stroke.^2,21^ Some studies have revealed an association between PSD and neurological deficits, indicating that PSD may be a partial psychological reaction to, e.g. cognitive impairment, motor deficits (e.g. hemiplegia) and activities of daily living.^121,122^ This view is challenged by studies conceptualizing PSD as a neurobiological consequence rather than an adjustment disorder. For example, stroke patients have been reported to be at a three to four times higher risk for developing depression than orthopaedic patients or traumatic brain injury patients with comparable impairments or lesion volumes.^123,124^ Singh *et al.*^125^ identified both lesions in inferior frontal regions and functional impairment in activities of daily living assessed 1-month post-stroke to predict PSD development, yet functional impairment was the strongest predictor. To the best of our knowledge, no previous study has investigated the relationship between specific depressive symptoms like motivational and emotional deficits or anxiety and stroke severity (NIHSS). Importantly, our findings suggest no association between these specific depressive symptom domains and stroke severity in the acute stage post-stroke. Thus, our findings align with the notion that PSD symptoms primarily depend on anatomical causes rather than functional impairments. Nevertheless, our sample included patients in a very early stage after stroke who participated in an early rehabilitation programme and were embedded in frequent multidisciplinary therapies. As we did not evaluate PSD symptoms and stroke severity in later stages post-stroke, functionally impaired patients might develop increased PSD symptoms after discharge when they are confronted with impairments and drawbacks in their everyday life. Due to our eligibility criteria for early rehabilitation treatment, our study sample contained more severely affected patients than the average stroke population.^64^ Furthermore, 55.5% of our patients showed at least mild depressive symptoms already in the acute stage after stroke,^63,126^ which is substantially higher than the average prevalence of ∼30% at any time up to 5-year post-stroke.^3^ Thus, we investigated the neuroanatomical correlates of PSD in a large sample of strongly impaired and prevalently depressive stroke patients. Accordingly, identifying the underlying pathophysiological mechanisms of PSD symptoms and potential risk factors in an average stroke population remains important to confirm our findings, identify patients at risk and individualize PSD prevention and treatment.

While our current results support a symptom-specific view of anatomical correlates of PSD, depressive symptoms in MD have been suggested to arise from different risk factors and biomarkers.^127,128^ Thus, while our findings may not be generalized to MD, they can inform future research on symptom-specific neural mechanisms underlying MD.

Crucially, the presence of a lesion does not necessarily indicate an increased risk for depression. Recent research by Trapp *et al.*^35^ conducted a large-scale LSM study on depression after focal brain damage and revealed that certain lesions can actually reduce the likelihood of developing depressive symptoms, i.e. exhibiting resilience to the manifestation of depressive symptoms. Especially, brain regions associated with the default mode network were identified as regions of resilience. These findings highlight an interesting factor to look at in the future of LSM.^35^ As another outlook for future studies, it may be very promising to look at individual depressive symptom clusters from a functional and structural network perspective. This could be achieved by integrating normative connectome data and examining correlations with canonical resting state networks, which could potentially unveil network-level pathological mechanisms underlying PSD.

## Conclusion

This relatively large-scale study reveals crucial aspects of the aetiology of PSD by showing that distinct depressive symptoms (i.e. motivational symptoms, emotional symptoms, cognitive symptoms, somatic symptoms and anxiety) in the acute stage post-stroke are related to specific lesion sites. These results extend the understanding of the aetiology and pathophysiology of depression and the underlying functional and anatomical networks. Furthermore, we provide essential evidence of symptom-specific lesion lateralization in PSD, with symptoms of anxiety specifically being hemispheric. Our findings suggest that PSD arises from localized neural symptom clusters and does not solely represent a mere psychological adaptation following the functional impairment after stroke. Considering that stroke and thus PSD are life-changing events with a substantial impact on the patient's health, multivariate approaches to lesion–symptom mapping can reveal specific therapeutic targets for future interventions individually fitted to specific symptoms in post-stroke patients, thereby promoting optimal rehabilitative outcomes.

## Supplementary Material

fcad275_Supplementary_Data

## Data Availability

Derived anonymized data supporting the findings of this study, and resulting signature region of interest masks, are available upon reasonable request.
