# Supplementary material for: Neuroanatomy of post-stroke depression: the association between symptom clusters and lesion location
Source: Brain Commun. 2023 Oct 25;5(5):fcad275. doi: 10.1093/braincomms/fcad275 (PMC10613857; doi:10.1093/braincomms/fcad275)
Supplement: fcad275_Supplementary_Data [file fcad275_supplementary_data.docx]

# Supplementary material

## MRI protocols and scan parameters

Stroke patients in the sample were scanned in the clinical routine over five years using three different MRI scanners. Following MRI protocols were used to assess diffusion-weighted imaging (DWI) and Fluid-attenuated inversion recovery (FLAIR) scans: First scanner Philips 1.5T: DWI: TR = 4126ms, TE = 95ms, voxel size 1.8mm (r-l) x 2.99mm (a-p) x 6mm, min. gap = 0.8mm, 22 to 24 axial slices. FLAIR: TR = 6000ms, TE = 100ms, voxel size 1.38mm (r-l) x 1.1mm (a-p) x 4mm, min gap = 0.8mm, 36 to 40 axial slices. Second scanner Philips 3T: DWI: TR = 3425ms, TE = 73ms, voxel size 1.5mm (r-l) x 2.19mm (a-p) x 5mm, min. gap = 0.5mm, min. 30 axial slices. FLAIR: TR = 8000ms, TE = 135ms, voxel size 1mm (r-l) x 0.65mm (a-p) x 4mm, min gap = 1mm, min. 33 axial slices. Third scanner Philips 1.5T: DWI: TR = 4421ms, TE = 110ms, voxel size 1.6mm (r-l) x 2.55mm (a-p) x 5mm, min. gap = 0.5mm, min. 28 axial slices. FLAIR: TR = 9000ms, TE = 120ms, voxel size 1mm (r-l) x 0.8mm (a-p) x 4mm, min gap = 0.5mm, min. 28 axial slices.

## Lesion symptom mapping

For our analyses, the MATLAB toolbox for the SVR-LSM^1^, derived from the implementations for SVR-LSM by Zhang *et al*.^2^, was used. It includes new features like a graphical user interface, parallel processing to speed up analyses, and a new way of removing lesion volume bias from both lesion and behavioral data. Furthermore, it uses the MATLAB Statistics and Machine Learning Toolbox implementation. We used the recommended default hyperparameters with a cost of 30, Sigma of 0.45, and Epsilon of 0.10.^2^

## Five-fold cross-validation with ten replicates was used. The training was accomplished with 20% of the data left out for testing. During training, voxels were used as features, and the behavioral score was the training target. Each of the ten five-fold-cross-validation cycles resulted in SVR-LSM parametric 𝛽-maps. These were derived from the non-linear back projection from the infinite feature space to the original brain space to infer regional symptom-lesion relationships. The final 𝛽-map was derived from the average of the ten single 𝛽-maps generated during cross-validation. This map included raw 𝛽-values for each voxel, representing the strength of the relationship to the variable under examination before statistical testing. Of note, this multivariate approach of SVR-LSM has been extensively validated and has since then been used in various studies.^3–7^ After permutation testing, results were voxel-wise thresholded at *p* < 0.005, and resulting *p*-maps were smoothed using a 2mm Gaussian smoothing kernel. Lastly, lesions were projected on the ch2better template in MRIcron^8^.

##
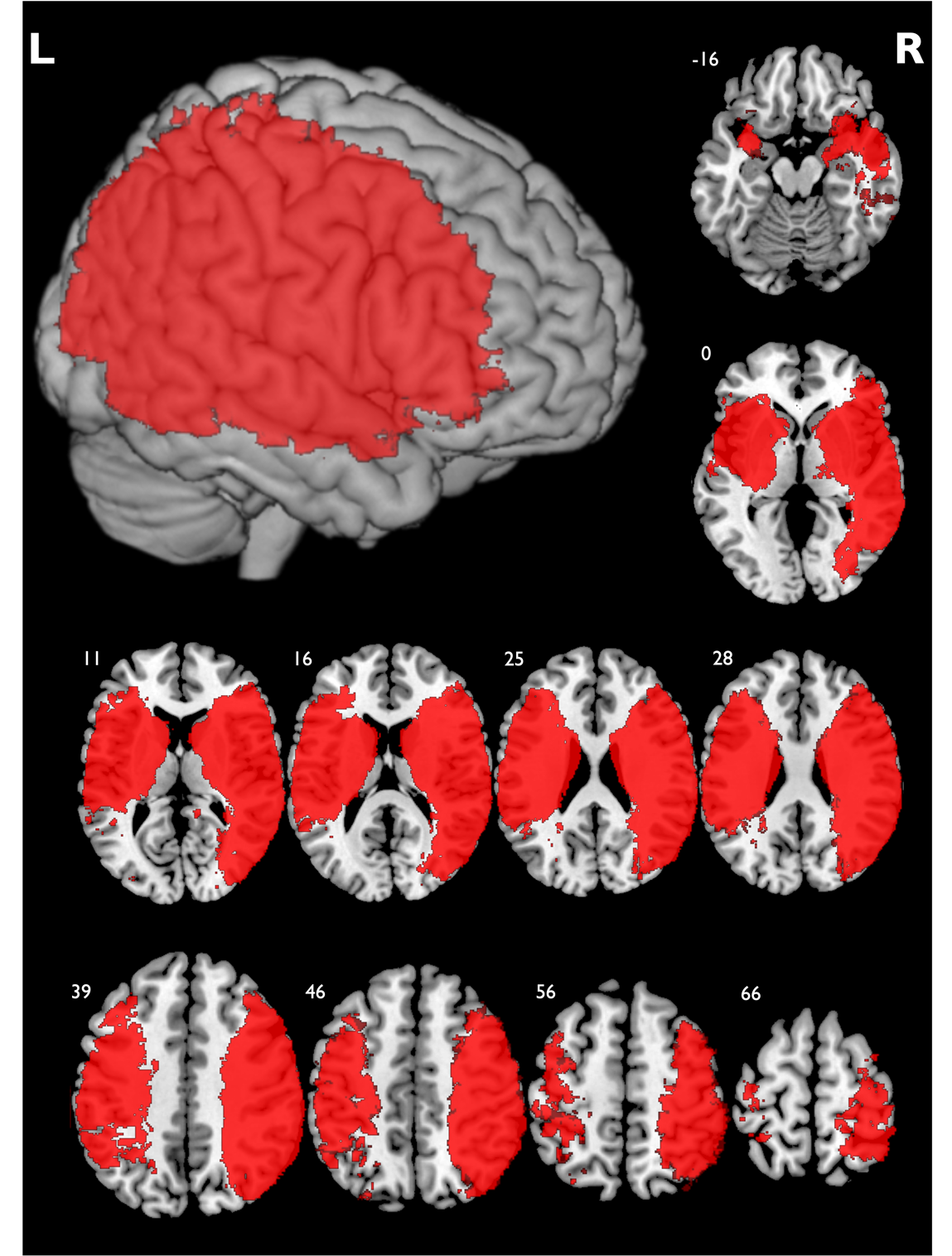


**Supplementary Figure 1 Minimum lesion overlap map.** Lesion overlap map of patients included in the SVR-LSM analyses displayed with the minimum lesion threshold of n ≥ 5 overlapping lesions. Coordinates indicate corresponding z-value in Montreal Neurological Institute (MNI) space. Please note, that small overlap into ventricles is due to co-registration for display in MRIcron. L, left; R, right.

**
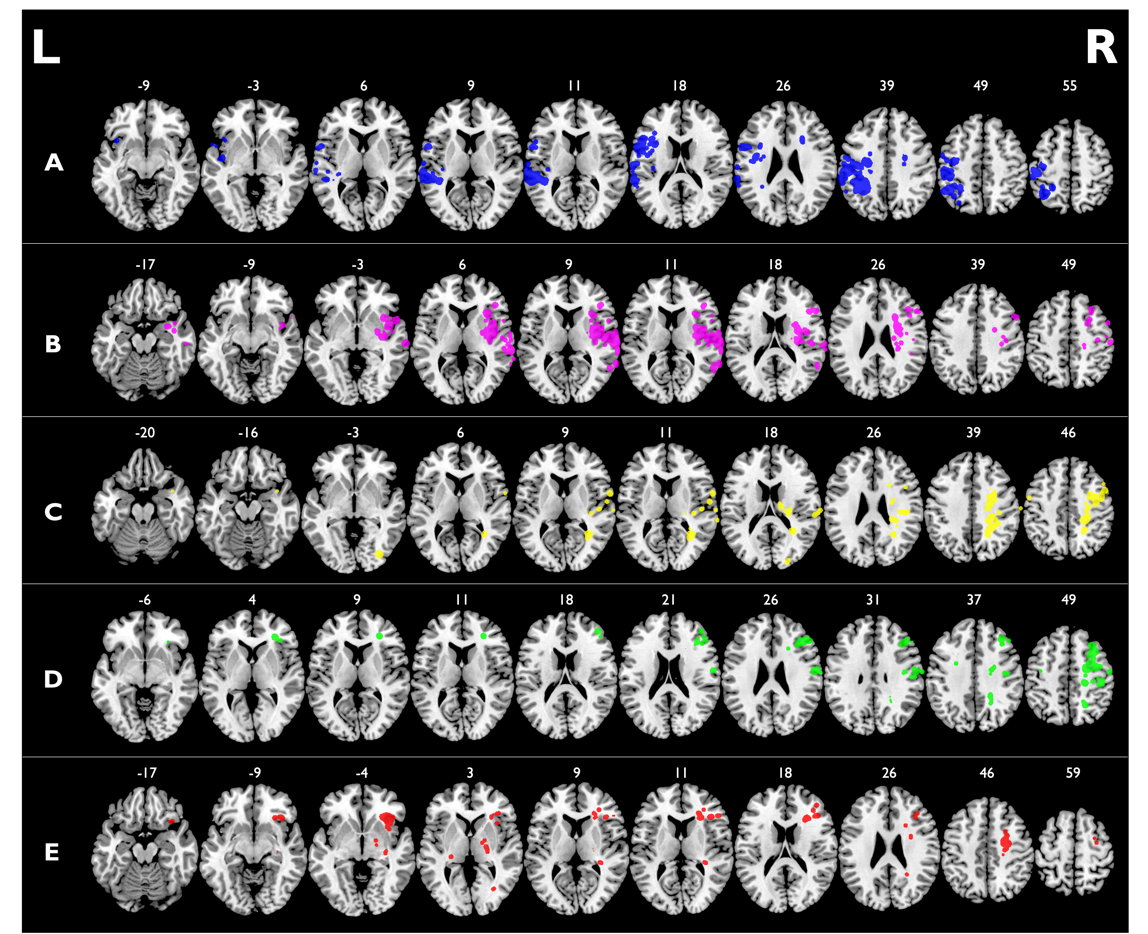
**

**Supplementary Figure 2 SVR-LSM results and lesion location associations of depressive symptom domains mapped individually.** SVR-LSM results and lesion location associations of depressive symptom domains based on the conceptual-empirical classification with a voxel-wise threshold set to p < 0.005 (n = 200). Results were smoothed using a 2mm isotropic Gaussian smoothing filter. Coordinates indicate corresponding z-value in Montreal Neurological Institute (MNI) space. **(A)** Anxiety. **(B)** Somatic. **(C)** Emotional. **(D)** Cognitive. **(E)** Motivational. L, left; R, right.

## PCA analysis

PCA with oblique rotation was used to analyze MADRS item scores by extracting five factors. The analysis results with factor loadings > 0.5 are presented in Supplementary Table 1. Except for suicidal thoughts (highest loading 0.44 on Factor 5), all items had significant factor loadings of >0.5, supporting the differentiation between the five factors. The goal of this additional data-driven approach was to receive five factors, which are clinically and item-wise concurring with the symptom domains derived in the conceptual-empirical approach. Thus, we expected to find a clinically related item structure in the five factors representing the depressive symptom domains of the conceptual-empirical approach. Both classification approaches showed an item-wise high correspondence. For example, the items ‘reduced sleep’ and ‘reduced appetite’ were attributed to ‘somatic symptoms’, and, accordingly, showed a high loading on Factor 2. Likewise, emotional symptoms of sadness were unambiguously classified together in Factor 3. When comparing conceptual-empirical and data-driven symptom domains, items representing the cognitive domain slightly differed between both approaches. In the data-driven approach, ‘concentration deficits’ represented a single Factor 4, whereas ‘pessimistic thoughts’ loaded high on Factor 5 together with ‘lassitude’. Also, Factor 1 consisted predominantly of ‘inner tension’ and the item ‘inability to feel’, which was categorized as a motivational symptom in the conceptual-empirical rating approach. Notably, the items ‘lassitude’ and ‘inability to feel’, commonly considered motivational symptoms in the conceptual categorization, were related to different factors in the data-driven categorization. The item ‘suicidal thoughts’ was not well categorized by the PCA, failing to have significant factor loadings in any of the factors. This may be because patients indicated very few ‘suicidal thoughts’ in the interview (mean = 0.27) with relatively low variance (±0.788), as well as a high skewness (3.347) and kurtosis (11.160) compared to the other MADRS items. Thus, in this sample, the symptom could not be well represented in a factor.

In general, extracted factors in the PCA yielded factor loadings corresponding to the five depressive symptom domains formed in the conceptual-empirical classification approach. Likewise, the conceptual-empirical and data-driven classification approaches revealed strikingly similar lesion regions associated with the five specific depressive symptom domains, i.e., five factors (Supplementary Table 1, Supplementary Fig. 1). Please note that the aim of this paper was not primarily to compare two different classification methods. Instead, with the data-driven approach, we intended to support the results of our conceptual-empirical classification and wanted to show the robustness of a heterogeneous lesion-symptom association in PSD.

| **Items** |  | **Apparent**  **sadness** | **Reported**  **sadness** | **Innter**  **Tension** | **Reduced**  **Sleep** | **Reduced**  **appetite** | **Concentration**  **Deficits** | **Lassitude** | **Inability**  **to feel** | **Pessimistic**  **thoughts** | **Suicidal**  **thoughts** |
| --- | --- | --- | --- | --- | --- | --- | --- | --- | --- | --- | --- |
| **Apparent sadness** | R_Sp_ | 1 |  |  |  |  |  |  |  |  |  |
|  | p (2-sided) |  |  |  |  |  |  |  |  |  |  |
| **Reported sadness** | R_Sp_ | 0.438 | 1 |  |  |  |  |  |  |  |  |
|  | p (2-sided) | <.001 |  |  |  |  |  |  |  |  |  |
| **Inner**  **tension** | R_Sp_ | 0.297 | 0.308 | 1 |  |  |  |  |  |  |  |
|  | p (2-sided) | <.001 | <.001 |  |  |  |  |  |  |  |  |
| **Reduced**  **sleep** | R_Sp_ | 0.002 | 0.182 | 0.216 | 1 |  |  |  |  |  |  |
|  | p (2-sided) | 0.978 | 0.01 | 0.002 |  |  |  |  |  |  |  |
| **Reduced**  **appetite** | R_Sp_ | 0.19 | .339 | 0.092 | 0.27 | 1 |  |  |  |  |  |
|  | p (2-sided) | 0.007 | <.001 | 0.194 | <.001 |  |  |  |  |  |  |
| **Concentration deficits** | R_Sp_ | 0.106 | 0.126 | 0.132 | 0.101 | 0.1 | 1 |  |  |  |  |
|  | p (2-sided) | 0.134 | 0.075 | 0.062 | 0.154 | 0.16 |  |  |  |  |  |
| **Lassitude** | R_Sp_ | 0.364 | 0.231 | 0.175 | 0.011 | 0.085 | 0.183 | 1 |  |  |  |
|  | p (2-sided) | <.001 | <.001 | 0.013 | 0.876 | 0.234 | 0.01 |  |  |  |  |
| **Inability to feel** | R_Sp_ | 0.2 | 0.206 | 0.328 | 0.079 | 0.029 | 0.246 | 0.326 | 1 |  |  |
|  | p (2-sided) | 0.004 | 0.003 | <.001 | 0.263 | 0.684 | <.001 | <.001 |  |  |  |
| **Pessimistic thoughts** | R_Sp_ | 0.199 | 0.286 | 0.231 | 0.125 | 0.145 | 0.15 | 0.183 | 0.239 | 1 |  |
|  | p (2-sided) | 0.005 | <.001 | <.001 | 0.078 | 0.04 | 0.034 | 0.01 | <.001 |  |  |
| **Suicidal**  **thoughts** | R_Sp_ | 0.312 | 0.367 | 0.252 | 0.057 | 0.137 | 0.192 | 0.433 | 0.43 | 0.269 | 1 |
|  | p (2-sided) | <.001 | <.001 | <.001 | 0.424 | 0.052 | 0.007 | <.001 | <.001 | <.001 |  |

**Supplementary Table 1 Inter-item correlations between the individual items of the MADRS interview**

| **Items** | **Factor 1** | **Factor 2** | **Factor 3** | **Factor 4** | **Factor 5** |
| --- | --- | --- | --- | --- | --- |
| Apparent sadness |  |  | -0.809 |  |  |
| Reported sadness |  |  | -0.740 |  |  |
| Inner tension | 0.820 |  |  |  |  |
| Reduced sleep |  | 0.849 |  |  |  |
| Reduced appetite |  | 0.517 |  |  |  |
| Concentration deficits |  |  |  | 0.953 |  |
| Lassitude |  |  |  |  | 0.562 |
| Inability to feel | 0.581 |  |  |  |  |
| Pessimistic thoughts |  |  |  |  | 0.874 |
| *Eigenvalues* | *3.248* | *1.233* | *1.014* | *0.867* | *0.835* |
| *Cumulative variance (%)* | *32.483* | *44.818* | *54.960* | *63.633* | *71.985* |

**Supplementary Table 2 MADRS factor analysis, including factor loadings >0.5 after oblique rotation.**

| **Components** | **Eigenvalue** | **Explained variance (%)** | **Cumulative variance (%)** |
| --- | --- | --- | --- |
| 1 | 3.248 | 32.483 | 32.483 |
| 2 | 1.233 | 12.334 | 44.818 |
| 3 | 1.014 | 10.142 | 54.960 |
| 4 | 0.867 | 8.673 | 63.633 |
| 5 | 0.835 | 8.352 | 71.985 |
| 6 | 0.713 | 7.133 | 79.119 |
| 7 | 0.653 | 6.529 | 85.648 |
| 8 | 0.547 | 5.466 | 91.114 |
| 9 | 0.459 | 4.589 | 95.703 |
| 10 | 0.43 | 4.297 | 100,000 |

**Supplementary Table 3 MADRS factor analysis, overview of total variance explained per component**


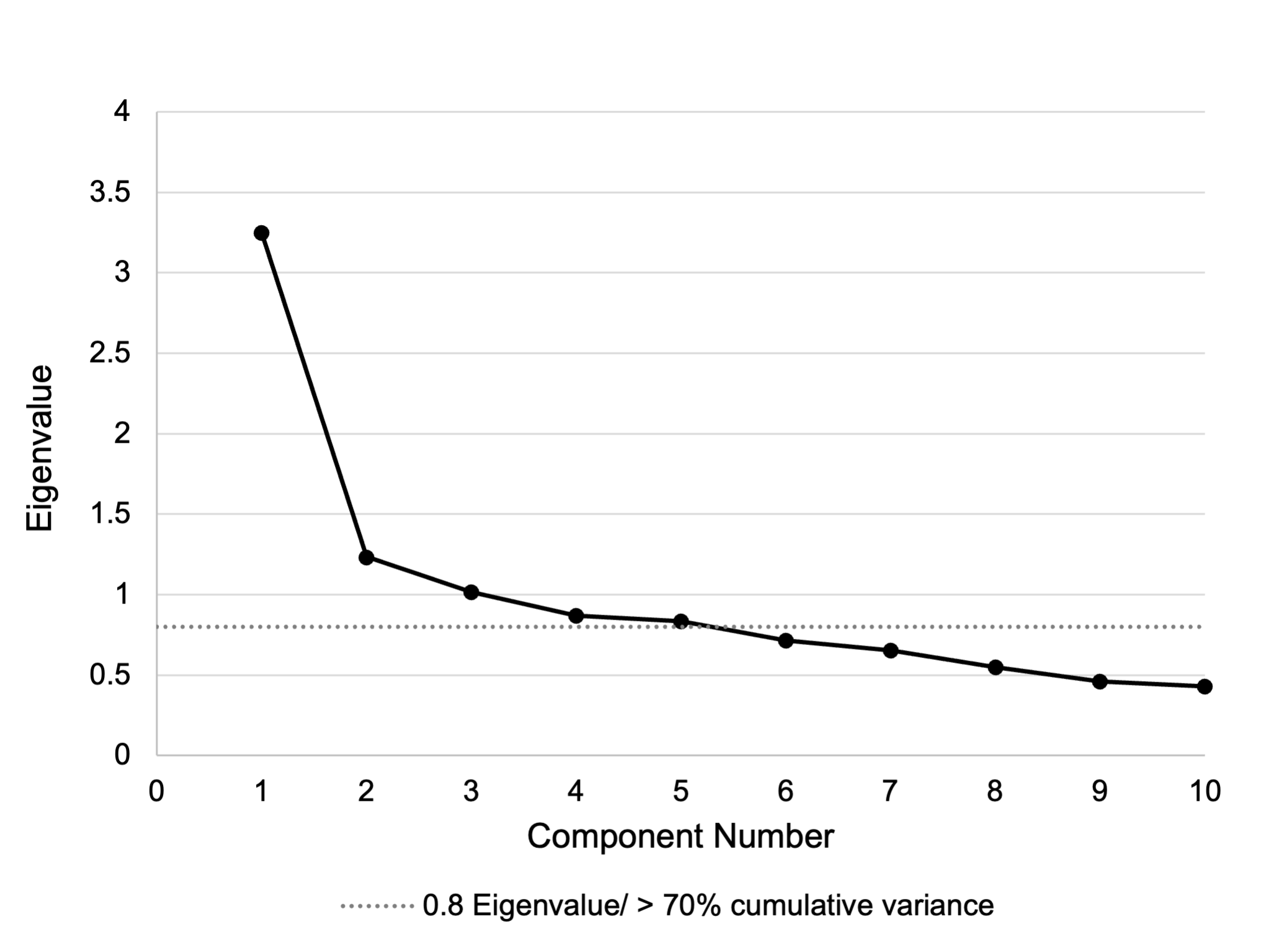


**Supplementary Figure 3 Scree plot of the PCA showing the derived factors with the corresponding Eigenvalues**

| **Brain region** | **Factor 1** | **Factor 2** | **Factor 3** | **Factor 4** | **Factor 5** |
| --- | --- | --- | --- | --- | --- |
| inferior frontal gyrus | L | R |  | R |  |
| middle frontal gyrus | L | R |  | R (dlPFC) | R |
| superior frontal gyrus |  | R |  | R | R |
| insula | L | R | R (av) |  | R (a) |
| precentral gyrus | L | R | R | R | R |
| postcentral gyrus | L | R | L/R | R |  |
| middle temporal gyrus |  | R |  |  |  |
| superior temporal gyrus | L | R | R |  |  |
| inferior parietal lobe | L | R | R |  | R |
| superior parietal lobe | L | R | R | R |  |
| amygdala |  | R |  |  |  |
| frontal operculum | L | R |  |  |  |
| central operculum | L | R | L/R |  |  |
| parietal operculum | L | R | R |  |  |
| putamen |  | R |  |  | R |
| pallidum |  | R |  |  | R |
| temporal pole | L | R |  |  |  |
| thalamus | L |  |  |  |  |
| orbitofrontal cortex |  |  |  |  | R |
| lateral occipital cortex |  |  | R | R |  |
| frontal pole |  |  |  | R |  |
| pons | L |  |  |  | L |

**Supplementary Table 4 SVR-LSM results of data-driven categorization.** L(eft) or R(ight) indicate the hemisphere with significant clusters of voxels (*p* < 0.005) in a given brain region. Classification of anatomical structures was performed using the Harvard-Oxford Cortical and Subcortical structural atlases. dlPFC, dorsolateral prefrontal cortex; av, anterior-ventral; a, anterior.


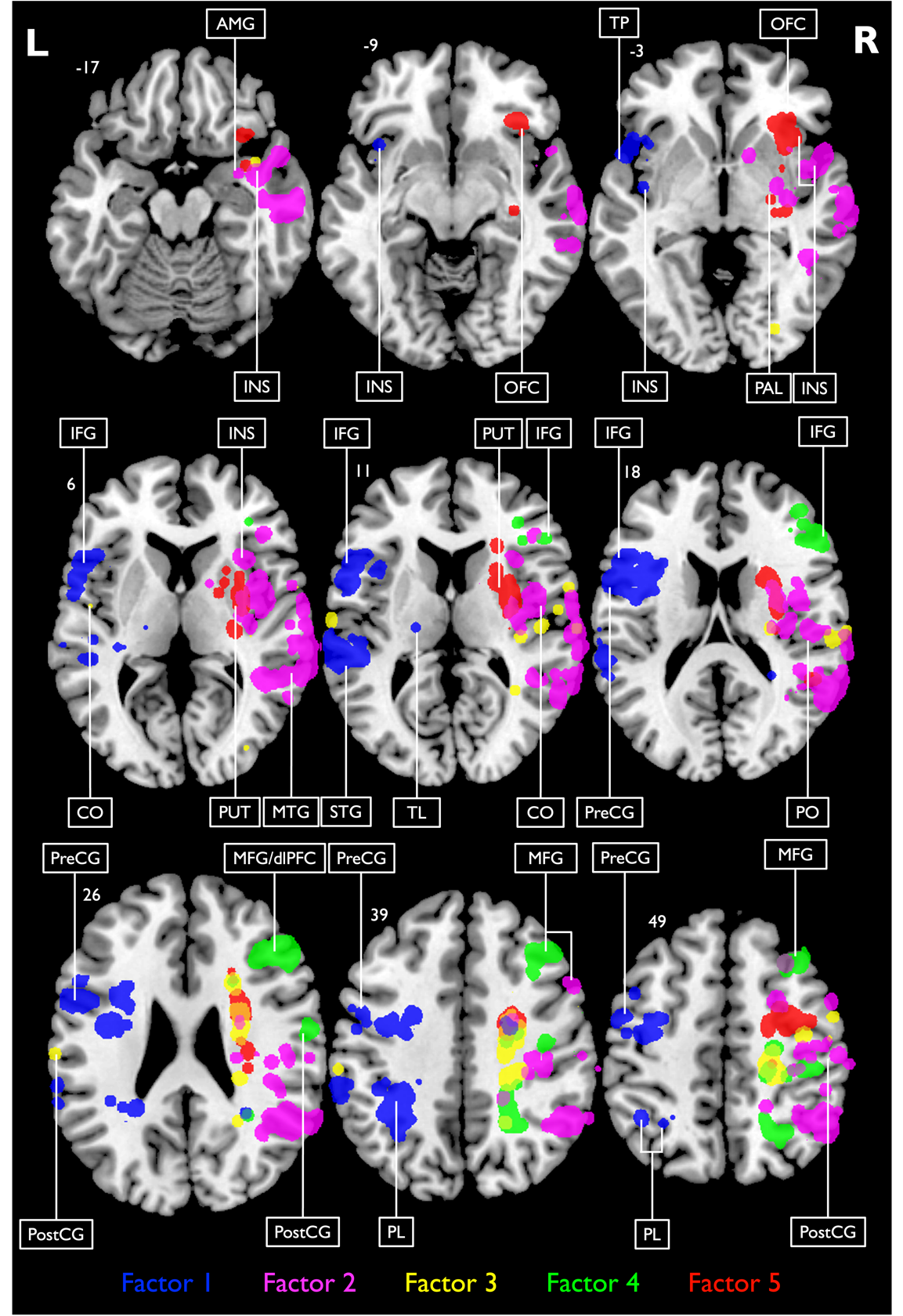


**Supplementary Figure 4 SVR-LSM results of the data-driven approach.** Voxel-wise threshold was set to *p* < 0.005 (*n* = 200). Results were smoothed using a 2mm Gaussian smoothing filter. Coordinates indicate corresponding z-value in Montreal Neurological Institute (MNI) space. Blue: Factor 1. Pink: Factor 2. Yellow: Factor 3. Green: Factor 4. Red: Factor 5. Please find the clinical interpretation of the five factors in the supplementary text. Classification of anatomical structures was performed using the Harvard-Oxford Cortical and Subcortical structural atlases. Predominant clusters are marked in white. AMG, amygdala; INS, insula; OFC, orbitofrontal cortex; TP, temporal pole; PAL, pallidum; PUT, putamen; IFG, inferior frontal gyrus; MTG, middle temporal gyrus; CO, central operculum; PO, parietal operculum; TL, thalamus; STG, superior temporal gyrus; PL, parietal lobe; PreCG, precentral gyrus; PostCG, postcentral gyrus; dlPFC, dorsolateral prefrontal cortex; MFG, middle frontal gyrus. L, left; R, right.

## Supplementary references

1. DeMarco AT, Turkeltaub PE. A multivariate lesion symptom mapping toolbox and examination of lesion-volume biases and correction methods in lesion-symptom mapping. *Hum Brain Mapp*. 2018;39(11):4169-4182.

2. Zhang Y, Kimberg DY, Coslett HB, Schwartz MF, Wang Z. Multivariate lesion-symptom mapping using support vector regression. *Hum Brain Mapp*. 2014;35(12):5861-5876.

3. Lacey EH, Skipper-Kallal LM, Xing S, Fama ME, Turkeltaub PE. Mapping Common Aphasia Assessments to Underlying Cognitive Processes and Their Neural Substrates. *Neurorehabil Neural Repair*. 2017;31(5):442-450.

4. Zhao L, Biesbroek JM, Shi L, et al. Strategic infarct location for post-stroke cognitive impairment: A multivariate lesion-symptom mapping study. *J Cereb Blood Flow Metab*. 2018;38(8):1299-1311.

5. Wiesen D, Sperber C, Yourganov G, Rorden C, Karnath HO. Using machine learning-based lesion behavior mapping to identify anatomical networks of cognitive dysfunction: Spatial neglect and attention. *Neuroimage*. 2019;201:116000.

6. Garcea FE, Greene C, Grafton ST, Buxbaum LJ. Structural Disconnection of the Tool Use Network after Left Hemisphere Stroke Predicts Limb Apraxia Severity. *Cereb Cortex Commun*. 2020;1(1):1-20.

7. Ghaleh M, Lacey EH, Fama ME, Anbari Z, Demarco AT, Turkeltaub PE. Dissociable Mechanisms of Verbal Working Memory Revealed through Multivariate Lesion Mapping. *Cereb Cortex*. 2020;30(4):2542-2554.

8. Rorden C, Karnath HO, Bonilha L. Improving Lesion-Symptom Mapping. *J Cogn Neurosci*. 2007;19(7):1081-1088.
